# Enhancing postpartum hemorrhage prediction in pernicious placenta previa: a comparative study of magnetic resonance imaging and ultrasound nomogram

**DOI:** 10.3389/fphys.2023.1177795

**Published:** 2023-08-08

**Authors:** Zixuan Song, Pengyuan Wang, Lue Zou, Yangzi Zhou, Xiaoxue Wang, Tong Liu, Dandan Zhang

**Affiliations:** ^1^ Department of Obstetrics and Gynecology, Shengjing Hospital of China Medical University, Shenyang, China; ^2^ Department of Radiology, Shengjing Hospital of China Medical University, Shenyang, China; ^3^ Department of Health Management, Shengjing Hospital of China Medical University, Shenyang, China

**Keywords:** pernicious placenta previa, postpartum hemorrhage, LASSO analysis, clinical prediction model, nomogram

## Abstract

**Objective:** To explore the risk factors of postpartum hemorrhage (PPH) in patients with pernicious placenta previa (PPP) and to develop and validate a clinical and imaging-based predictive model.

**Methods:** A retrospective analysis was conducted on patients diagnosed surgically and pathologically with PPP between January 2018 and June 2022. All patients underwent PPP magnetic resonance imaging (MRI) and ultrasound scoring in the second trimester and before delivery, and were categorized into two groups according to PPH occurrence. The total imaging score and sub-item prediction models of the MRI risk score/ultrasound score were used to construct Models A and B/Models C and D. Models E and F were the total scores of the MRI combined with the ultrasound risk and sub-item prediction model scores. Model G was based on the subscores of MRI and ultrasound with the introduction of clinical data. Univariate logistic regression analysis and the logical least absolute shrinkage and selection operator (LASSO) model were used to construct models. The receiver operating characteristic curve andision curve analysis (DCA) were drawn, and the model with the strongest predictive ability and the best clinical effect was selected to construct a nomogram. Internal sampling was used to verify the prediction model’s consistency.

**Results:** 158 patients were included and the predictive power and clinical benefit of Models B and D were better than those of Models A and C. The results of the area under the curve of Models B, D, E, F, and G showed that Model G was the best, which could reach 0.93. Compared with Model F, age, vaginal hemorrhage during pregnancy, and amniotic fluid volume were independent risk factors for PPH in patients with PPP (*p* < 0.05). We plotted the DCA of Models B, D, E, F, and G, which showed that Model G had better clinical benefits and that the slope of the calibration curve of Model G was approximately 45°.

**Conclusion:** LASSO regression nomogram based on clinical risk factors and multiple conventional ultrasound plus MRI signs has a certain guiding significance for the personalized prediction of PPH in patients with PPP before delivery.

## Introduction

The concept of pernicious placenta previa (PPP) was first proposed by Chattopadhyay et al., 1993 (Chattopadhyay et al., 1993) to describe placenta previa in a woman who had undergone a cesarean section and whose placenta attachment point happened to be the site of the uterine scar. Approximately 50% of patients with PPP are complicated with placenta accreta spectrum (PAS), which is prone to uncontrollable hemorrhage during labor and postpartum, endangering the safety of mother and child (Zhu et al., 2021). Due to the revision of the family planning policy in China, cesarean section, induced abortion, and other intrauterine surgery are increasing. Consequently, the number of patients with PPP and the incidence of PAS are gradually increasing and have become one of the important causes of maternal death (Chen et al., 2016; Jiang et al., 2019). According to a study, the risk of placenta accreta in women with a previous cesarean section is 35 times higher than that in those without (Yu et al., 2016). Therefore, it is advocated to use early prenatal diagnosis to diagnose PPP in clinical practice to reduce maternal and fetal mortality (Hou et al., 2020).

Ultrasound and magnetic resonance imaging (MRI) are commonly used in prenatal diagnosis; however, some controversies still exist in the diagnostic results and accuracy of the two methods (Riteau et al., 2014). Ultrasonography is a traditional examination method that is simple to operate, reusable, and low-cost. Particularly, Doppler ultrasound can detect the blood flow of the placenta, retroplacental space, and blood sinus (Mar et al., 2015; Silver and Barbour, 2015). However, its clinical application is limited due to its low sensitivity (Judson et al., 2008), especially in case of placental villus invasion, intestinal gas, bladder filling, and depends on blood vessel size, flow rate, and the experience level of the operator, which may lead to misdiagnosis or missed diagnosis in some patients. In addition, placenta previa without placenta accreta can also form lacunae under ultrasound, which can be misdiagnosed as placental adhesion (Silver and Barbour, 2015).

Magnetic resonance imaging (MRI) is a multi-planar imaging technique where the signal generated by the resonance of the nucleus in a strong magnetic field is reconstructed. It has the characteristics of no angle limitation, high soft tissue resolution, large field of view, and it is less influenced by placental position, intestinal gas, maternal body size, and other factors. Therefore, it can clearly show the placenta’s shape and position in the case of oligohydramnios or the placenta located in the posterior wall of the uterus. Particularly, it can clearly visualize the uterine-placenta interface, placenta and muscular layer, parametrical tissue, and adjacent pelvic organs (Zaghal et al., 2019; Arthuis et al., 2021). In addition, MRI includes many imaging modalities highlighting different tissue contrasts, such as T1-weighted imaging (T1WI), T2-weighted imaging (T2W1), and diffusion-weighted imaging (DWI) sequences, which can accurately reflect the anatomical relationship between the placenta and the uterus, evaluate the hemodynamic changes in the microvessels, and provide numerous imaging information for diagnosis (Ueno et al., 2014). However, due to objective factors such as the high cost and complexity of MRI examinations, MRI cannot be widely applied in the diagnosis of implanted PPP.

Presently, studies on prenatal diagnosis of PPP are still active in obstetrics locally and internationally, and the corresponding reports are endless. However, objective indicators are inadequate to accurately determine and evaluate the “degree of PPP,” which leads in some cases to underestimating the difficulty and risk of the operation. Inadequate preoperative preparation can cause excessive intraoperative hemorrhage, ureteral and bladder injury, fatal hemorrhage that requires removing the uterus, and severe maternal death. In some other cases, the risk of hemorrhage is overestimated or may involve surrounding organs. Therefore, preventive measures such as preset balloon occlusion of the artery and placement of ureteral stents can impose unnecessary medical risks on patients and also cause waste of medical resources (Huo et al., 2021). Furthermore, the leading cause of maternal morbidity and mortality worldwide is *postpartum* hemorrhage (PPH) (Say et al., 2014). It has been reported that adequate preoperative preparation and multidisciplinary team management can reduce the amount of intraoperative or postoperative hemorrhage in patients with PPP (He et al., 2019; Ozdemir et al., 2021; Zhou et al., 2021).

Accurate prenatal diagnosis and assessment of the degree and extent of placenta accreta in PPP are crucial for selecting obstetric surgery, particularly for preventing the risk of massive hemorrhage during surgery. Therefore, to improve the evaluation rate of intraoperative hemorrhage in patients with PPP, this study proposes to construct a clinical prediction model based on the clinical risk factors of PPP combined with preoperative ultrasound and MRI examination to predict the risk of intraoperative hemorrhage. Through the intuitive, concise, and convenient expression of the nomogram, the possibility of hemorrhage during PPP can be predicted as early as possible, which can effectively ensure the safety of mother and child, avoid excessive medical treatment, allow complete preparation before surgery, and prevent adverse events.

## Methods

### Ethics statement

This retrospective study was performed in accordance with thelaration of Helsinki and the Strengthening the Reporting of Observational Studies in Epidemiology (STROBE) statement. Ethics approval was granted by the Human Research Ethics Committee at the Shengjing Hospital of China Medical University (Ethics Code: 2022PS132K).

### Patients selection

**FIGURE 1:**
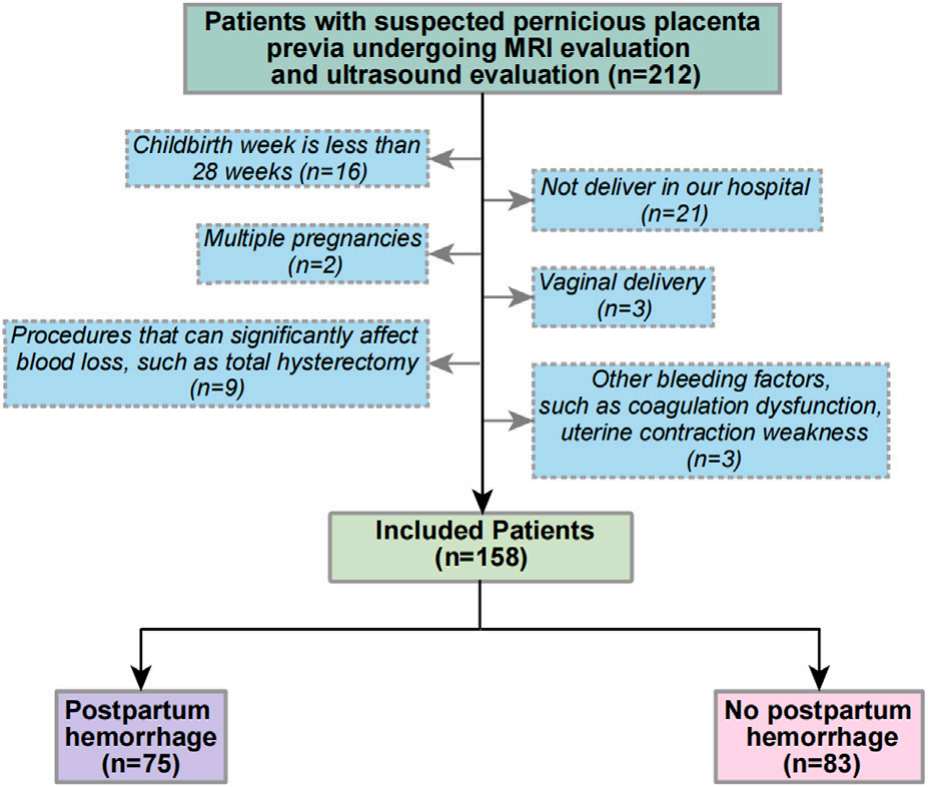
The flowchart of patient selection.

A retrospective cohort from January 2018 to June 2022 was developed in Shengjing Hospital of China Medical University. All patients were suspected of having PPP due to routine ultrasound examination during pregnancy and received MRI scoring of PPP in the second trimester and ultrasound examination and scoring before delivery. The exclusion criteria were (Chattopadhyay et al., 1993): gestational age of delivery < 28 weeks; (Zhu et al., 2021): non-delivery in our hospital (Jiang et al., 2019); multiple pregnancies (Chen et al., 2016); vaginal delivery (Yu et al., 2016); perioperative procedures that significantly affect blood loss, such as abdominal aortic balloon occlusion and total hysterectomy; and (Hou et al., 2020) the evidence of other hemorrhage factors, such as coagulopathy and uterine contraction weakness, among others. Figure 1 shows the flow chart of patient selection criteria.

### Color-Doppler ultrasound examination

Color-Doppler ultrasound examination was performed using the GE Voluson E8/E10. The frequency of the probe was set at 3.6–5.0 MHz. Next, the patient was placed in a supine position with full bladder, and the abdomen was continuously examined. Subsequently, the fetus and other structures were carefully observed. According to the PPP ultrasound scale, the review signs including the location of the placenta, placental thickness, continuity of the clear space, bladder line, placental lacunae, condition of subplacental vascularity, and cervical morphology in the cervical sinus, and also the number of previous cesarean delivery were evaluated (Chen et al., 2021). Transvaginal sonography (TVS) was performed to identify the cervical canal, the internal meatus, and the relationship between the placental margin and the internal meatus when the placenta was not evident on abdominal examination after the patient was asked to urinate. The inferior uterine wall and the bladder interface were also evaluated.

### Magnetic resonance imaging examination

A Philips Ingenia 3.0 T superconducting magnetic resonance imager (Ingenia, Philips Healthcare, Best, Netherlands) equipped with 4- and 8-channel body-phased coils and a built-in 3T MR system was used in this study. Scanning the patient with a full bladder ensured that the bladder floor and walls could be more clearly presented. The patients entered the scanner bore feet first and in the supine or lateral position. Magnetization-prepared turbo field echo (T1-TFE), turbo spin echo (T2-TSE), and T2-TSE-SPectral Attenuated Inversion Recovery (SPAIR) sequences were selected to perform coronal, sagittal, and axial scans, respectively. T1-TFE sequence parameters were: repetition time (TR) 10.13 m, echo time (TE) 2.30 m, slice thickness 8 mm, and pitch 1 mm; T2-TSE sequence parameters were: TR 1100 m, TE 75 m, slice thickness 8 mm, pitch 1 mm, a field of view (FOV) 450 × 398 mm, and matrix 312 × 241. T2-TSE-SPAIR was used for fat suppression (Bour et al., 2014; Ueno et al., 2016).

### Diagnostic criteria for PPP

The diagnosis of PPP was based on the comprehensive diagnosis of surgery and pathology. Surgical criteria included having a previous cesarean section, placenta previa pregnancy, the placenta attached to the original uterine scar, placental thickening, and difficulty in manually removing the placenta after delivery. There were multiple “placental lacunae” in different shapes and sizes in the placenta, and uterine hemorrhage was uncontrollable. Pathological diagnosis was defined as abnormal adhesion of the placenta to the myometrium or chorionic tissue in the smooth muscle of the uterus.

### Data collection

The clinical data of patients with suspected PPP were collected through the Hospital Information System, including the patient’s age, neonatal weight, gestational age, and parity. Using a picture archiving and communication system (PACS), the MRI and ultrasound images in the second trimester and before delivery, respectively, were reviewed by two experienced radiologists/sonographers (PW and LZ) independently, and the images were scored according to the MRI and ultrasound risk scores. When there was disagreement, a third physician participated (TW) in the discussion and reached a consensus. The MRI and ultrasound scales were previously developed and validated by our academic group (Zou et al., 2022) (Table1; Table 2). Intraoperative blood loss was measured by weight, visual acuity, shock index, and estimated blood loss. PPH in this study was defined as blood loss of ≥1,000 mL within 24 h after cesarean section.

**TABLE 1:** MRI-based scoring system for pernicious placenta previa.

MRI characteristics	0	1	2
Placenta position	Normal	Marginal placenta previa or low lying placenta	Complete placenta previa
Placental/uterine bulge	Normal	Suspected	Yes
Placental heterogeneity	None	Suspected	Yes
T2-dark bands in placenta	None	1 place	≥2 places
Abnormal intraplacental vascularity	None	1 place	≥2 places
Abnormal vascularization of the placental bed	None	1 place	≥2 places
Loss of T2 hypointense interface	Normal	Suspected	Yes
Bladder wall interruption	Normal	Blurring	Interruption
Penetrating placenta implantation	None	Suspected	Yes
Myometrial thinning and interruption	Normal	Thickness ≤ 1 mm	Interruption
Number of previous cesarean deliveries	-	1	≥2

**TABLE 2:** Ultrasound-based scoring system for pernicious placenta previa.

Ultrasonic characteristics	0	1	2
Placenta position	Normal	Marginal placenta previa or low lying placenta	Complete placenta previa
Placental thickness (cm)	< 3 cm	3–5 cm	>5 cm
Loss of clear zone	Continuity	Local interruption	Disappeared
Bladder line	Continuity	Local interruption	Disappeared
Placental Lacunae	None	Present	Fused with boiling water sign
Condition of the subplacental vascularity	Normal blood flow	The blood flow increased, forming a cluster	The emergence of “cross-border” blood vessels
Cervical blood sinus	None	Present	Fused with boiling water sign
Cervical morphology	Complete	Incomplete	Disappeared
Number of previous cesarean deliveries	-	1	≥2

### Construct and evaluate different prediction models and nomograms

The risk factors related to PPH were selected to construct different prediction models, which are shown in Table 3. Models A and C are the sums of the risk scores of ultrasound and MRI; Models B and D were the prediction models established by ultrasound and MRI sub-item scores using logistics regression; Model E was the total score of MRI plus ultrasound risk score; and Model F was the prediction model constructed by ultrasound plus MRI risk factors sub-item scores using logistics regression. Model G was based on Model F plus clinical data to establish a prediction model. Due to the limited items in the clinical data, we used univariate analysis to screen the risk factors for PPH. However, MRI and ultrasound risk scores included many sub-scores; therefore, we used the least absolute shrinkage and selection operator (LASSO) analysis, which is more conducive for screening important variables from several variables.

**TABLE 3:** The construction methods of different models.

Prediction models	Selection of indicators for construct models
Model A	Total score of MRI risk score
Model B	Sub-item prediction model of MRI risk score
Model C	Total score of ultrasound risk score
Model D	Sub-item prediction mode of ultrasound risk score
Model E	Total score of MRI risk score + total score of ultrasound risk score
Model F	Sub-item prediction mode of MRI + ultrasound risk score
Model G	Important risk factor indicators in clinical data (by univariate logistic regression analysis)
	+ important risk factors in MRI and ultrasound Sub-item (by LASSO analysis)

To evaluate the discriminatory ability of the prediction model, receiver operating characteristic (ROC) curves were constructed, and area under the curve (AUC) values were calculated (Obuchowski and Bullen, 2018). The clinical application value of the model was determined through theision curve analysis (DCA) by quantifying the net benefit to the patient under different threshold probabilities (Vickers and Holland, 2021). According to the evaluation results of the different models, the model with the strongest predictive ability and the best clinical effect was selected to construct a nomogram. The nomogram was internally validated by bootstrapping (1,000 resampling) (Henderson, 2005).

### Statistical analysis

Data were analyzed in an R Studio environment using R (version 3.6.3; R Foundation for Statistical Computing, Vienna, Austria; http://www.r-project.org). To evaluate the risk factors associated with PPH in clinical data, univariate logistic regression analysis was performed on the clinical data, and odds ratios and 95% confidence intervals were calculated. Statistical significance was set as *p* < 0.05.

LASSO analysis was used to screen the MRI and ultrasound high-risk score items in Model G that may affect PPH. The logical LASSO model is a contraction method that actively selects from numerous variables that may be multicollinear in regression to produce a more relevant and interpretable set of predictive variables (Tibshirani, 1996). We use ten-fold cross-validation to select the penalty term λ. The built-in function of R produces two automatic λ that selects the smallest binomial deviation facilitating a more comprehensive study of the included covariables.

## Results

### Characteristics of patients

Overall, 212 patients received MRI and ultrasound risk scores in our center from January 2018 to June 2022 for suspected PPP, and 158 patients were included according to the exclusion criteria. Of these, 75 patients had PPH. Table 4 shows the specific patient characteristics.

**TABLE 4:** Characteristics of patients with pernicious placenta previa.

Characteristic	No postpartum hemorrhage N = 83	*Postartum* hemorrhage N = 75
Age (years old)		
< 35	67 (81%)	39 (52%)
≥35	16 (19%)	36 (48%)
Neonatal weight (g)		
< 2,500	28 (34%)	23 (31%)
2,500–4,000	53 (64%)	42 (56%)
>4,000	2 (2.4%)	10 (13%)
Childbirth week		
< 37	47 (57%)	43 (57%)
≥37	36 (43%)	32 (43%)
Number of pregnancy		
2	21 (25%)	22 (29%)
3	22 (27%)	17 (23%)
4	19 (23%)	11 (15%)
≥5	21 (25%)	25 (33%)
Number of abortion		
None	23 (28%)	26 (35%)
1–2	43 (52%)	34 (45%)
≥3	17 (20%)	15 (20%)
PIH		
None	81 (98%)	69 (92%)
Yes	2 (2.4%)	6 (8.0%)
GDM		
None	77 (93%)	64 (85%)
Yes	6 (7.2%)	11 (15%)
Time between previous cesarean section (years)		
1–5	35 (42%)	29 (39%)
6–10	35 (42%)	41 (55%)
>10	13 (16%)	5 (6.7%)
Vaginal hemorrhage during pregnancy		
None	35 (42%)	49 (65%)
Yes	48 (58%)	26 (35%)
Fetal position		
Cephalic presentation	63 (76%)	61 (81%)
Breech presentation	13 (16%)	12 (16%)
Transverse lie presentation	7 (8.4%)	2 (2.7%)
Amniotic fluid index		
< 5	10 (12%)	2 (2.7%)
5–18	73 (88%)	70 (93%)
>18	0 (0%)	3 (4.0%)
MRI risk score—Placenta position		
1	26 (31%)	10 (13%)
2	57 (69%)	65 (87%)
MRI risk score—Placental/uterine bulge		
0	62 (75%)	48 (64%)
1	20 (24%)	18 (24%)
2	1 (1.2%)	9 (12%)
MRI risk score—Placental heterogeneity		
0	27 (33%)	3 (4.0%)
1	35 (42%)	22 (29%)
2	21 (25%)	50 (67%)
MRI risk score—T2-dark bands in placenta		
0	25 (30%)	7 (9.3%)
1	34 (41%)	27 (36%)
2	24 (29%)	41 (55%)
MRI risk score—Abnormal intraplacental vascularity		
0	36 (43%)	20 (27%)
1	42 (51%)	30 (40%)
2	5 (6.0%)	25 (33%)
MRI risk score—Abnormal vascularization of the placental bed		
0	26 (31%)	14 (19%)
1	43 (52%)	34 (45%)
2	14 (17%)	27 (36%)
MRI risk score - Loss of T2 hypointense interface		
0	22 (27%)	6 (8.0%)
1	41 (49%)	25 (33%)
2	20 (24%)	44 (59%)
MRI risk score—Bladder wall interruption		
0	54 (65%)	39 (52%)
1	27 (33%)	28 (37%)
2	2 (2.4%)	8 (11%)
MRI risk score—Penetrating placenta implantation		
0	68 (82%)	38 (51%)
1	11 (13%)	20 (27%)
2	4 (4.8%)	17 (23%)
MRI risk score—Myometrial thinning and interruption		
0	38 (46%)	12 (16%)
1	41 (49%)	39 (52%)
2	4 (4.8%)	24 (32%)
MRI risk score—Number of previous cesarean deliveries		
1	74 (89%)	55 (73%)
2	9 (11%)	20 (27%)
Ultrasound risk score—Placenta position		
1	15 (18%)	3 (4.0%)
2	68 (82%)	72 (96%)
Ultrasound risk score—Placental thickness (cm)		
0	21 (25%)	7 (9.3%)
1	50 (60%)	44 (59%)
2	12 (14%)	24 (32%)
Ultrasound risk score—Loss of clear zone		
0	41 (49%)	7 (9.3%)
1	21 (25%)	13 (17%)
2	21 (25%)	55 (73%)
Ultrasound risk score—Bladder line		
0	42 (51%)	11 (15%)
1	27 (33%)	28 (37%)
2	14 (17%)	36 (48%)
Ultrasound risk score—Placental Lacunae		
0	57 (69%)	19 (25%)
1	16 (19%)	36 (48%)
2	10 (12%)	20 (27%)
Ultrasound risk score—Condition of the subplacental vascularity		
0	23 (28%)	2 (2.7%)
1	26 (31%)	17 (23%)
2	34 (41%)	56 (75%)
Ultrasound risk score—Cervical blood sinus		
0	75 (90%)	42 (56%)
1	7 (8.4%)	11 (15%)
2	1 (1.2%)	22 (29%)
Ultrasound risk score—Cervical morphology		
0	80 (96%)	45 (60%)
1	2 (2.4%)	19 (25%)
2	1 (1.2%)	11 (15%)
Ultrasound risk score—Number of previous cesarean deliveries		
1	74 (89%)	55 (73%)
2	9 (11%)	20 (27%)

PIH: pregnancy induced hypertension syndrome; GDM: gestational diabetes mellitus.

### Models construction and ROC and DCA curves for different models

**FIGURE 2:**
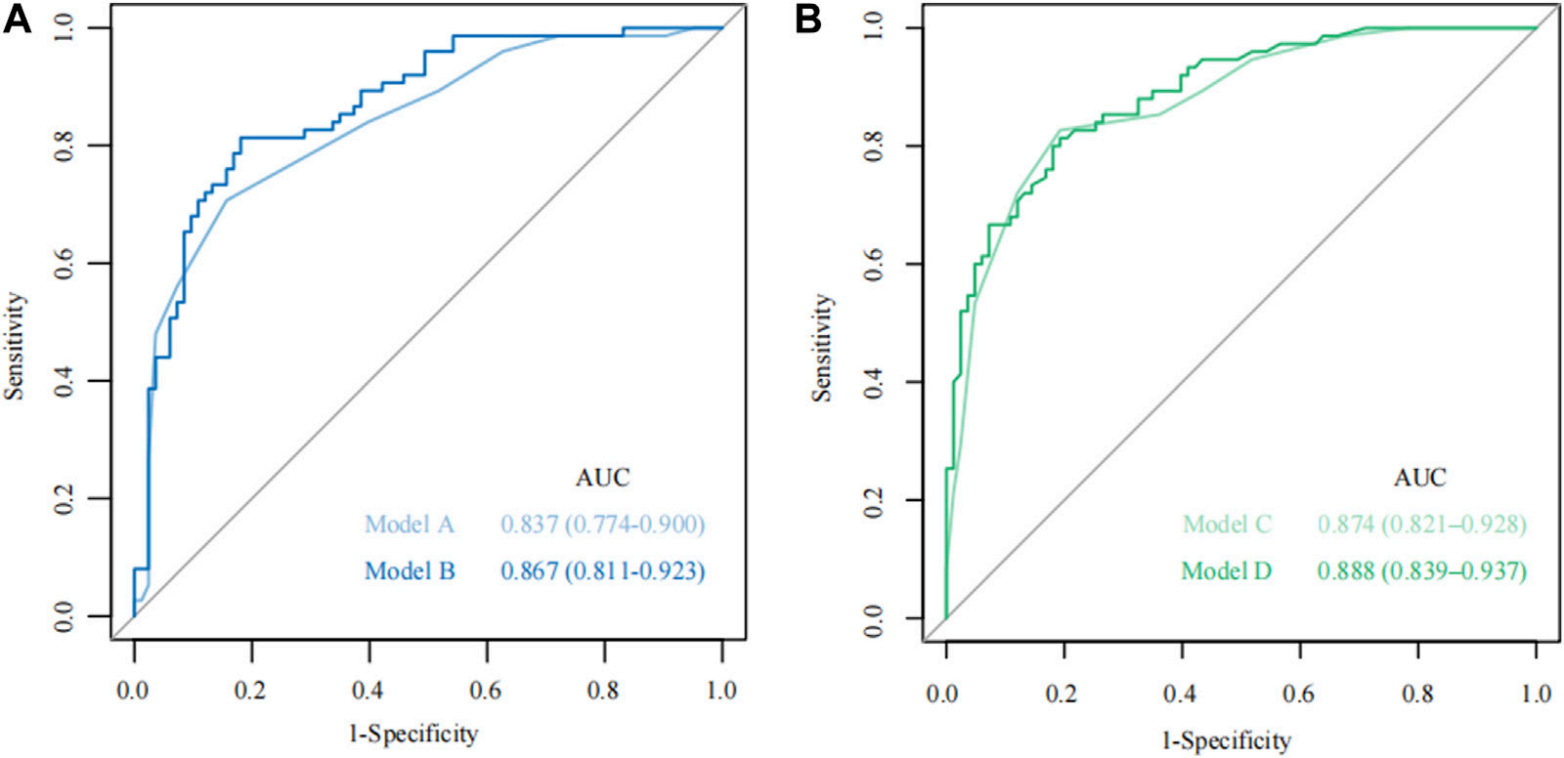
The receiver operating characteristic (ROC) curve for Model A-D. **(A)** The ROC curve of Models A and B; **(B)** The ROC curve of Models C and D.

**FIGURE 3:**
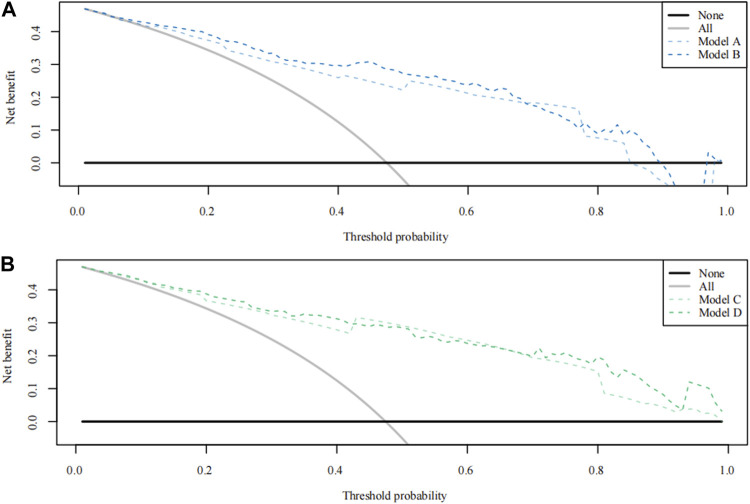
Theision curve analysis (DCA) curve for Model A-D. **(A)** The DCA curve of Models A and B; **(B)** The DCA curve of Model C and Model D.

According to the total score and logistics regression of the MRI risk score, Model A and B were constructed, respectively, and ROC and DCA curves were obtained (Figure 2A; Figure 3A). The results showed that the predictive ability and clinical benefit of Model B were better than that of Model A. Similarly, the ultrasound risk scoring models were constructed with Model C and D, respectively, and ROC and DCA curves were drawn (Figure 2B; Figure 3B). The results showed that model D had a better predictive ability and clinical benefit than Model C. The above results indicate that the logistics regression score model of MRI and ultrasound have a better predictive ability and clinical benefit than the total score in predicting PPH.

**FIGURE 4:**
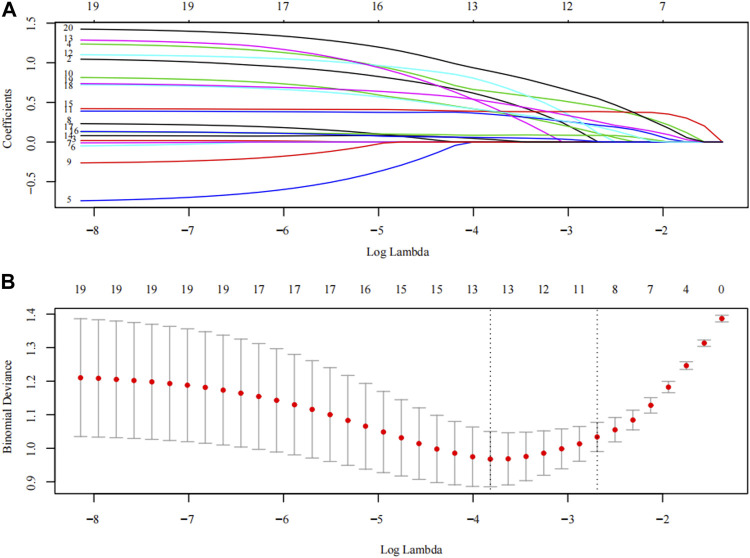
The LASSO analysis. The inflection point or curve trend in the graph can indicate the optimal degree of freedom or regularization level, and a lower Binomial Deviance value indicates a better fit of the model. **(A)** Plots for LASSO regression coefficients over different values of the penalty parameter; **(B)** The number of risk factors for the sub-score of MRI risk score and ultrasonic risk score was determined by cross-validation of penalty terms in LASSO analysis.

**FIGURE 5:**
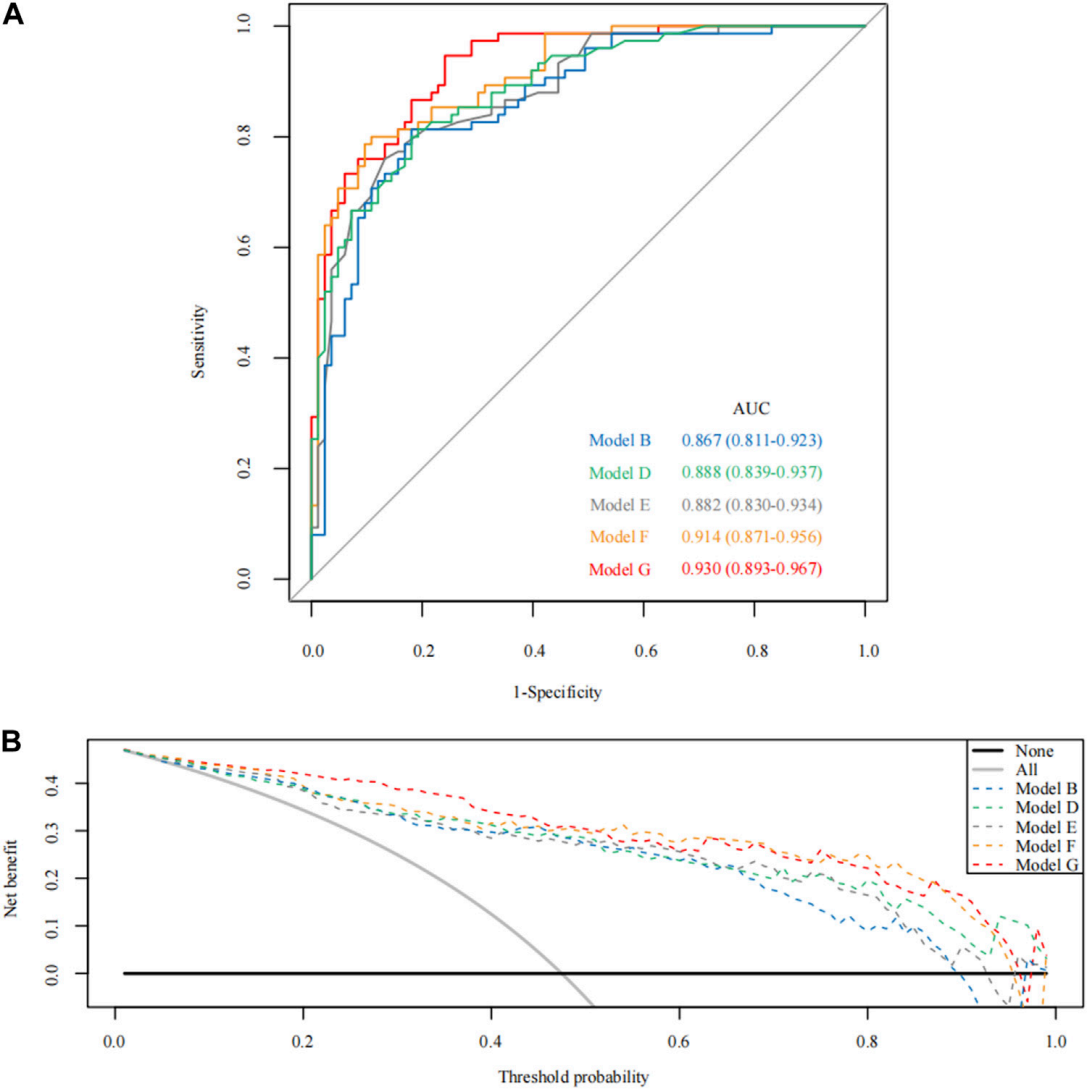
The receiver operating characteristic (ROC) curve and theision curve analysis (DCA) curve for Model B/D/E/F/G. **(A)** The ROC curve of Model B/D/E/F/G; **(B)** The DCA curve of Model B/D/E/F/G.

Models E and F were constructed using the total score and logistics regression of MRI with an ultrasound risk score. Model G was combined with MRI plus ultrasound risk factors and clinical data, and all data were screened for risk factors. Due to the limited clinical data items, univariate logistic regression analysis was used to screen the risk factors of PPH (Table 5). MRI and ultrasound risk were adopted for the LASSO analysis, which was more conducive to screening important variables in several variables to screen risk factors (Table 6; Figure 4). ROC curves and DCA curves of Models B, D, E, F, and G were drawn, respectively, and the results showed that Model G had the best predictive ability and clinical benefits compared with other Models (Figure 5).

**TABLE 5:** The univariable logistic regression analysis of postpartum hemorrhage.

Characteristic	Univariate logistic regression		
	OR	95% CI	*p*-value
Age (years old)	3.87	1.93, 8.02	< 0.001*
Neonatal weight (g)	1.52	0.89, 2.66	0.133
Childbirth week	0.97	0.52, 1.83	0.929
Number of pregnancy	1.03	0.79, 1.34	0.839
Number of abortion	0.86	0.55, 1.34	0.510
PIH	3.52	0.78, 24.6	0.131
GDM	2.21	0.79, 6.71	0.139
Time between previous cesarean section (years)	0.88	0.55, 1.42	0.601
Vaginal bleeding during pregnancy	0.39	0.20, 0.73	0.004*
Fetal position	0.69	0.38, 1.22	0.213
Amniotic fluid index	6.43	1.75, 41.4	0.015*

PIH: pregnancy induced hypertension syndrome; GDM: gestational diabetes mellitus; OR: odds ratio; CI: confidence interval; *: *p* < 0.05.

**TABLE 6:** The LASSO analysis of postpartum hemorrhage.

Characteristic	LASSO coefficient
MRI risk score—Placenta position	0.660
MRI risk score—Placental/uterine bulge	-
MRI risk score—Placental heterogeneity	0.637
MRI risk score—T2-dark bands in placenta	-
MRI risk score—Abnormal intraplacental vascularity	-
MRI risk score—Abnormal vascularization of the placental bed	-
MRI risk score—Loss of T2 hypointense interface	-
MRI risk score—Bladder wall interruption	-
MRI risk score—Penetrating placenta implantation	0.386
MRI risk score—Myometrial thinning and interruption	0.350
MRI risk score—Number of previous cesarean deliveries	0.745
Ultrasound risk score—Placenta position	0.445
Ultrasound risk score—Placental thickness (cm)	0.033
Ultrasound risk score—Loss of clear zone	0.386
Ultrasound risk score—Bladder line	0.086
Ultrasound risk score—Placental Lacunae	0.056
Ultrasound risk score—Condition of the subplacental vascularity	0.393
Ultrasound risk score—Cervical blood sinus	0.508
Ultrasound risk score—Cervical morphology	0.893

### Construct the nomogram

**FIGURE 6:**
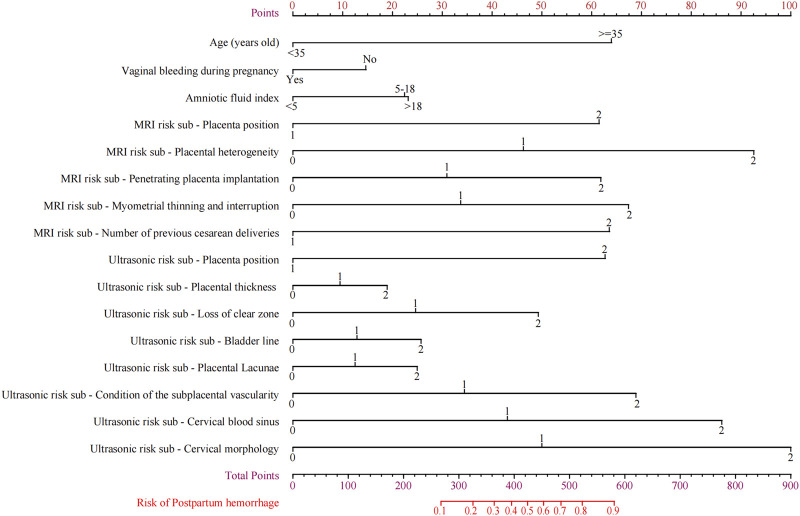
The nomograms of postpartum hemorrhage.

The nomogram that could be used to predict the PPH of PPP was constructed based on Model G (Figure 6). Reading and interpreting a nomogram for assessing PPH risk in PPP patients entails several steps (Chattopadhyay et al., 1993). Identify the variables representing risk factors and imaging scores on the axes of the nomogram (Zhu et al., 2021). Locate corresponding values and draw lines to the point scale (Jiang et al., 2019). Add up these points to obtain a total score, which determines the estimated probability of PPH. By drawing a line from the total points axis to the PPH probability axis, the likelihood of PPH occurrence can be interpreted at the intersection point. This process allows individualized risk assessment for PPP patients before delivery, aiding inision-making.

**FIGURE 7:**
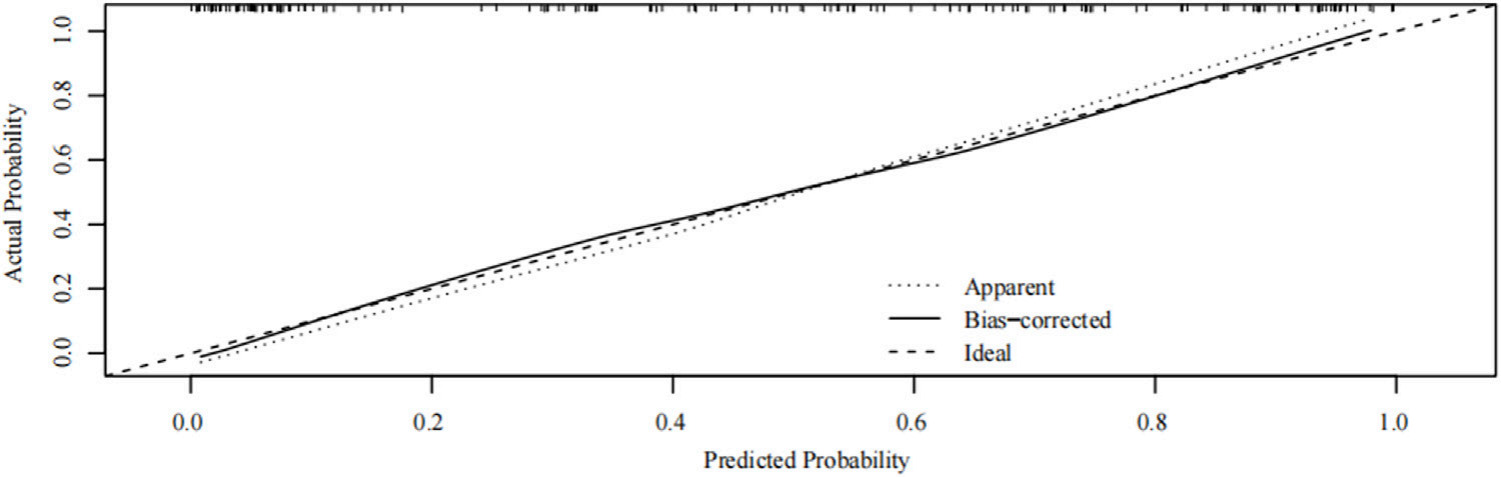
Internal verification plots of nomogram calibration curves by bootstrapping with 1,000 resamples.

Internal verification of the nomogram shows that the calibration curve was close to 45° (Figure 7), indicating that the predicted value agrees with the actual value.

## Discussion

### The main reason for PPH in patients with PPP is PAS

The primary cause of PPH in patients with PPP is placenta accreta syndrome PAS (Mehrabadi et al., 2015). The distribution of the muscular layer in the scar of the uterus is different from that in the normal part of the uterus due to cesarean section or surgery. During the second pregnancy, the placenta easily attaches to the scar of the uterus, which significantly increases the incidence ofidual dysplasia and placenta accreta (Silver and Barbour, 2015). Surgical injury to the endometrium may contribute to inadequate development ofidua basalis, further exacerbating the condition (McMahon et al., 1997). This leads to the expansion of the placenta towards the lower uterine segment and even the internal cervical orifice, resulting in placenta previa (Timor-Tritsch et al., 2019). The weak endometrial scar of the lower uterine segment allows the placenta previa to invade the muscular layer, potentially reaching the serosal layer and causing placenta accreta (Gurol-Urganci et al., 2011). In patients with placenta previa and PAS, the majority (90%) experience blood loss exceeding 3,000 mL during surgery, with some patients even exceeding 10,000 mL (Bonnar, 2000). Unfortunately, this severe bleeding can result in complications such as hemorrhagic shock, disseminated intravascular coagulation (DIC), multiple organ failure, hysterectomy, infertility, and even death if not effectively managed (Li P. et al., 2021).

To predict the likelihood of PPH during placenta previa, we constructed a prediction model based on six sub-independent risk factors: age, history of cesarean section, vaginal hemorrhage during pregnancy, amniotic fluid index, and ultrasound and MRI scores. The nomogram we developed indicated age to be a crucial imaging factor. Using 35 years as the cutoff, the prediction model score increased significantly by 65 points, indicating a higher risk of PPH in patients with placenta previa. Pregnancy hemorrhage and amniotic fluid index were subsequently identified as significant factors. Placental villi invading the myometrium results in a stronger attachment surface that reduces the likelihood of hemorrhage in the later stages of pregnancy (Bartels et al., 2018). As the placental villi penetrate deeper, an interconnecting network of blood vessels forms near the myometrium, which can lead to an increase in amniotic fluid quantity in some cases (Jha et al., 2018).

### Role of ultrasound signs in preoperative prediction of PPP

The role of ultrasound signs in the preoperative prediction of placenta percreta previa (PPP) has been extensively studied and plays a crucial role in patient management. According to the 2005 Royal College of Obstetricians and Gynaecologists guidelines, routine ultrasound screening at 20 weeks of gestation should determine the placenta position, and follow-up imaging should be performed if abnormalities are detected (Jauniaux et al., 2021). For patients with a history of cesarean section and uterine surgery, it is recommended to perform an MRI examination and three-dimensional Doppler ultrasound follow-up monitoring if the placenta is found to be in the anterior wall and reaching the internal cervical opening (Masselli et al., 2008). Presently, ultrasound diagnosis is also recognized as the preferred method for placenta accreta diagnosis. The sensitivity, specificity, and positive predictive value of placenta accreta were 87%–95%, 76%–98%, and 82%–93%, respectively (Chalubinski et al., 2013; D'Antonio and Bhide, 2014). In this study, all eight ultrasound imaging scores from the previously validated ultrasound score table were incorporated into the prediction model (Chen et al., 2021).

Distinct sonographic features indicative of PPP with placenta accreta include increased blood flow in the placenta (Collins et al., 2016), disordered blood flow signal distribution at the base of the placenta, colorful blood flow suggesting vascular distortion and aliasing, and “bridging” vessels between the placenta and uterine muscle wall. High-speed blood flow spectra and abnormal color blood flow signals at the placenta-uterine-bladder interface have been found to have high accuracy in diagnosing placenta accreta, with sensitivity and specificity ranging from 88% to 98% (Chou et al.; Wong et al., 2008). The clear space between the posterior placenta and the uterine muscle wall, known as the retroplacental venous plexus, is another ultrasound sign with diagnostic value (Comstock et al., 2004). Its disappearance or destruction indicates poorer accuracy of diagnosing placenta accreta compared to other ultrasound signs. However, when combined with color-Doppler ultrasound, the sensitivity increases to 90% (Bao Yajun and Wu, 2014; Jauniaux and Bhide, 2017; Jauniaux et al., 2018; D'Antonio et al., 2013; Yang et al., 2006).

Placental abnormal lacuna is a recognized ultrasound sign strongly associated with abnormal placentation. The presence of lacunae significantly increases the positive prediction of placenta accreta and is positively correlated with severe complications during and after the operation (Guy et al., 1990; Baughman et al., 2008; Doumouchtsis and Arulkumaran, 2010; Calì et al., 2013; Comstock and Bronsteen, 2014). The continuity of the bladder wall and the thickness of the lower uterine segment are additional specific signs for diagnosing PPP with placenta accreta (Comstock and Bronsteen, 2014). The interruption of the “bladder line” and thinning of the myometrium at the implantation site are indicative of bladder invasion and are associated with high diagnostic value. However, the sensitivity and specificity of myometrial thinning vary across studies (Twickler et al., 2000; Calì et al., 2013; Pilloni et al., 2016).

### Role of MRI signs in preoperative prediction of PPP

MRI offers advantages over ultrasound, such as higher sensitivity and specificity, excellent soft tissue and spatial resolution, wide imaging field, absence of ionizing radiation, and independence from maternal factors. Various MRI signs have been identified for diagnosing and evaluating PPP (Palacios Jaraquemada and Bruno, 2005). Different MRI signs have their advantages and disadvantages in the diagnosis of PAS. Practically, it is necessary to combine multiple signs to improve diagnostic accuracy. The low signal on T2WI (Derman et al., 2011) and true-fast imaging with steady-state precession (Tru-FISP) at the basal placental surface are specific signs of adhesive placenta. Localized expansion of placenta tissue and uterus, placental bulge, and bladder “tent sign” are associated with placenta percreta (Alamo et al., 2013; Jha et al., 2019). A localized bulge of the placenta and uterus indicates invasive placenta accreta. Combining multiple MRI signs improves diagnostic accuracy (Familiari et al., 2018; Li Q. et al., 2021). In MRI, the perfusion Intravoxel Incoherent Motion (IVIM) parameters fractional perfusion and diffusion also help differentiate different microvascular formation patterns in intrauterine growth restriction and assist in detecting subtle microvascular injury (Antonelli et al., 2022). The two-perfusion model also can provide complementary information to Intravoxel Incoherent Motion model parameters that may be useful in identifying placenta impairment (Maiuro et al., 2023).

In 2016, Ueno et al. (Ueno et al., 2016) developed the first prediction model of placenta accreta based on MRI signs. Six MRI signs were scored separately, and a scale was established with a score of 1–5. Statistical results showed that the MRI scoring system had a good diagnostic efficiency for PAS. Delli et al. (Delli Pizzi et al., 2019) included eight MRI signs of PAS and used a 5-point scale to construct an MRI scoring model, which showed high value in the diagnosis of PAS and prediction of adverse clinical outcomes. The scoring model based on MRI signs can quantitatively diagnose placenta accreta, although the two studies mentioned above excluded relevant clinical risk factors, and the scoring method was relatively complex and subjective; therefore, its practical application is limited. In contrast, this study included clinical measures after screening and also included five MRI imaging signs. Compared with MRI prediction alone, ultrasound features appeared to have a higher weight.

Advancements in radiomics have contributed to prenatal imaging diagnosis, therapeutic effect prediction, and prognostic evaluation (Chen et al., 2019; Romeo et al., 2019). Texture quantification analysis and machine learning techniques show high diagnostic value in identifying PPP. Deep learning methods combining radiological and depth features are promising for segmenting the placenta and determining implantation type (Xuan et al., 2021). Nomograms incorporating MRI morphology, radiomics features, and prenatal clinical factors achieve superior diagnostic performance for predicting PPP (Peng et al., 2022).

### Strengths and limitations

The strength of this study is that clinical risk factors, MRI signs in the second trimester, and ultrasound signs before delivery were included simultaneously. Logistic regression analysis was used to control confounding factors and facilitate a more comprehensive evaluation. The nomogram for predicting PPH in PPP based on the high-risk parameters had high accuracy (AUC = 0.930), and the calibration curve showed that the predicted probability of the model also agrees with the actual probability. The nomogram can visually show the scores corresponding to multiple independent risk factors of PPH in patients with PPP, and the probability of occurrence can be predicted by simple addition operation, which provides a simpler, visualized, and effective auxiliary method for predicting PPH and is easy to popularize and apply in clinical practice.

This study had some limitations. First, this was a single-center retrospective study with limited cases, which may have impacted the diagnostic efficacy of the model. Second, there was no external validation; therefore, the accuracy, repeatability, and value of the model in clinical practice require further verification. Moreover, our study utilized an 8 mm slice thickness due to adherence to customary clinical requirements of retrospective data, which has inherent limitations for radiological research, and future prospective studies can employ thinner slice thicknesses to improve imaging quality.

## Conclusion

The LASSO regression nomogram established in this study was simple to visualize based on clinical risk factors and multiple conventional ultrasounds plus MRI signs. Therefore, it provides a simple and practical tool for obstetricians to evaluate and predict the risk of PPH in patients with PPP, select appropriate surgical methods to reduce intraoperative hemorrhage, ensure the safety of pregnant women during delivery, and improve the prognosis of patients. Furthermore, prospective, multi-center, and large-sample studies are expected in the future to verify and improve the prediction model.

## Data Availability

The raw data supporting the conclusion of this article will be made available by the authors, without undue reservation.
